# Adipose-derived cellular therapies prolong graft survival in an allogenic hind limb transplantation model

**DOI:** 10.1186/s13287-021-02162-7

**Published:** 2021-01-29

**Authors:** Jingting Chen, Yinmin Wang, Haoyue Hu, Yao Xiong, Shoubao Wang, Jun Yang

**Affiliations:** 1 Department of Plastic and Reconstructive Surgery, Shanghai Ninth People’s Hospital, Shanghai Jiao Tong University School of Medicine, Shanghai, China; 2 Department of Plastic and Reconstructive Surgery, Shanghai General Hospital, School of Medicine, Shanghai Jiao Tong University, Shanghai, China; 3 Basic Medical School , Jining Medical University , Jining, 272000 China

**Keywords:** Vascularized composite allograft (VCA), Stromal vascular fraction (SVF), Immune modulation

## Abstract

**Background:**

The long-term survival after vascularized composite allotransplantation (VCA) is often limited by systemic rejection as well as the adverse effects of immunosuppressants. The stromal vascular fraction (SVF) can be expanded to produce adipose-derived stem cells (ADSC) which represents a combination of endothelial cells, preadipocytes, immune cells, and ADSC. It has been demonstrated that ADSC possess consistently reliable clinical results. However, literature is scarce regarding SVF in VCA. This study seeks to determine the impact of ex vivo allograft pretreatment in combination with SVF cells in the ability to promote composite tissue allotransplantation immunotolerance.

**Methods:**

A rat hind limb allotransplant model was used to investigate the influence of ex vivo pretreatment of SVF and ADSC on VCA survival. Intravascular cell-free saline, ADSC, or SVF was infused into the models with immunosuppressants. The histopathological examination and duration that the allografts went without displaying symptoms of rejection was documented. Peripheral T lymphocytes and Tregs were quantified with flow cytometry while allotissue expressions of CD31 were quantified with immunohistochemical staining (IHC). ELISA was used to detect vascular endothelial growth factor (VEGF)-A as well as anti- and pro-inflammatory cytokines.

**Results:**

We demonstrated that ex vivo treatment of allografts with SVF or ADSC prolonged allograft survival in contrast to medium control cohorts. There were also enhanced levels of immunomodulatory cytokines and increased VEGF-A and CD31 expression as well as reduced infiltration and proliferation of T lymphocytes along with raised Treg expressions.

**Conclusion:**

These studies demonstrated that adipose-derived cellular therapies prolong graft survival in an allogenic hind limb transplantation model and have the potential to establish immunotolerance.

**Supplementary Information:**

The online version contains supplementary material available at 10.1186/s13287-021-02162-7.

## Background

Vascularized composite allotransplantation (VCA) is an exciting modality for amputees and patients with large volume soft tissue loss that is not amenable to conventional reconstructive surgeries. It has the ability to restore function and appearance of damaged tissues in these patients [1, 2]. The ability of this modality of treatment is limited by the requirement of multiple immunosuppressant medications to allow for the grafts to survive in the recipient’s body [3, 4]. It is therefore essential for newer therapies that are able to effectively suppress the alloreactive immune response, thus opening up this treatment method to a larger population.

Several studies have attempted to promote peripheral tolerance and to modulate the local inflammatory immune responses within the graft using cellular therapy [5, 6]. Multiple cells types have been used for immune modulation or suppression to treat a range of maladies [7, 8]. It has been reported that bone marrow-derived stromal cells (BMSC) are immunosuppressive and inhibit the proliferation of alloreactive T cells [9]. BMSC have been intensely researched as a treatment modality in several animal and clinical studies across a myriad of diseases [10–14]. A previous study in a rat model hind limb transplantation demonstrated that infused BMSC into the allografts prolonged survival of transplanted hind limbs [15].

Another therapy that is derived from the nonadipocyte portion of adipose tissue is known as stromal vascular fraction (SVF). In 2001, a significant population of adipose-derived mesenchymal stem cells (ADSC) were extracted from a SVF isolate after being allowed to culture for several days [16]. Like BMSC, ADSC possess potent regenerative and immunomodulatory abilities. Their accessibility and excellent safety profile make them a promising alternative to BMSC-based treatments [17]. Our group previously demonstrated the important adjuvant role of ADSC when used as a pretreatment for allografts to induce VCA immunotolerance [18]. Freshly isolated SVF cells also possess substantial regenerative and immunosuppressive properties [19, 20]. This isolate is comprised of various immunomodulatory cells, which include ADSC, pre-adipocytes, pericytes, lymphocytes, smooth muscle cells, macrophages, endothelial cells, and endothelial precursor cells [16, 21, 22]. As these cells demonstrate equivalent regenerative and immunomodulatory abilities, and are logistically much easier to procure, they offer a viable alternative to ADSC treatments. However, evidence of SVF with immunomodulatory potential is lacking in the field of VCA.

In this study, we researched the prevention of allograft rejection through a unique perspective. We researched the ability and underlying mechanisms that allow ADSC and SVF to modulate the immune system to prevent allograft rejection in a VCA model.

## Methods

### Animals and immunosuppressive agents

A rodent hind limb allotransplant model was employed to investigate the influence of adipose-derived cellular therapies on VCA survival. Shanghai Sippr-BK Laboratory Animal Co. Ltd. (Shanghai, China) supplied 16 male Lewis and 8 male Brown Norway rats weighing 200–250 g for this study. Two different recipients received 1 hind limb each from the same donor. Group I (*n* = 4) was the media control group that did not receive cell therapy. Allografts of group II (*n* = 6) received ADSC ex vivo treatment. Allografts of group III (*n* = 6) received SVF ex vivo treatment. All rats received 5 days of post-operative oral cyclosporine (Sigma Aldrich, USA) at a dose of 16 mg/kg. All animal procedures were approved by the ethics committee of Shanghai Ninth People’s hospital Affiliated to Shanghai Jiao Tong University, School of Medicine. This work was carried out in strict compliance to the guidelines of the Laboratory Animal Manual of the NIH Guide to the Care and Use of Animals.

### Stromal vascular fraction isolation

Recipient Lewis rats aged 4–6 weeks were subjected to extraction of armpit and inguinal subcutaneous adipose tissues. These samples were diced into tiny pieces before being exposed for 1 h in serum-free Dulbecco’s modified Eagle’s medium (DMEM; Hyclone, Logan City, UT) supplemented by 0.1% collagenase (NB4, Serva, France) at 37°C. Tissue residues were removed by filtering the cell through a 40-mm nylon filter mesh (BD Falcon, Bedford, MA). The filtrate was centrifuged, and cell pellets were resuspended in phosphate buffered saline (PBS; Hyclone Laboratories, Inc., Logan, UT).

### Isolation, expansion, and characterization of ADSC

Rat ADSC was extracted from inguinal adipose tissue of Lewis rats (4 to 6 weeks old). These samples were first digested by 0.1% collagenase (NB4, Serva, France) for 1 h under gentle agitation at 37 °C, before being filtered through a 40-μm nylon strainer (Falcon, Corning, USA). The resultant substrate was subjected to centrifugation before the cell pellets were resuspended in 10 cm^2^ culture plates at 37 °C, under a 5% CO_2_ atmosphere which contained low glucose complete Dulbecco’s Modified Eagle Medium supplemented with 100 U/mL penicillin and 100 mg/mL streptomycin (Gibco, Invitrogen, USA) and 10% fetal bovine serum (FBS; Sciencell, USA). Further studies utilized cell passages 2 through 4.

Flow cytometric analysis of the cell surface marker profile of ADSC was performed with a FACS Calibur flow cytometer (BD Biosciences, San Jose, CA, USA) using primary antibodies for CD 90, CD 45, and CD44 (BioLegend Inc., San Diego, USA). A differentiation medium (Cyagen Bioscience, Inc., Guangzhou, China) was used to induce adipogenesis, osteogenesis, and chondrogenesis. Oil red, alizarin red, and alcian blue stains were used to identify differentiated cells.

### Procedure of hind limb transplantation, ex vivo allograft engineering, and general observations

The rat hind limb transplantation model described by previous study was used [23]. All mice were anesthetized with isoflurane. Dissection of the femoral artery was done on the donor rat prior to amputation mid-thigh of the hind limb. Sixty units/mL heparinized saline (Sigma Aldrich, St. Louis, MO, USA) was used to flush the donor’s osteomyocutaneous flap before limb vasculature was perfused with 1 million ADSC or SVF cells in cold saline or cell-free saline alone, as previously described [4]. The Lewis rat receiver received similar operation procedures prior to transplantation of the donor hind limb onto the recipient. An 18-gauge needle was used as intramedullary rod to perform femoral osteosynthesis. 12-0 nylon sutures were used to anastomose femoral vessels under an operating microscope. Muscle and skin were closed with intermittent sutures using 3-0 nylon thread.

Recipient rats were housed in a plastic cage where they could walk freely. Mice were monitored for symptoms of acute organ rejection daily. The clinical experimental end point of vascularized composite allotransplantation rejection was determined to be the formation of entire donor skin area epidermolysis, desquamation, exudation, eschar formation, and necrosis.

### Histological analyses of transplant allografts

Skin and muscle biopsies were obtained from the transplanted hind limb on days 7 and 15 post-transplantation or upon the occurrence of clinical picture of rejection. All biopsies were first immersed for 24 h of 4% paraformaldehyde prior to being embedded in paraffin. Tissue blocks were then sectioned and stained with hematoxylin and eosin using standard procedures.

In order to label cells for CD31, tissue sections were first processes for heat-induced antigen retrieval in a 10 mM citrate buffer (pH 7.3) prior to incubation with anti-rat CD31 (cat no. GB13063; 1:200 dilution; Servicebio, Wuhan, China). A chromogenic substrate solution (Beyotime Institute of Biotechnology, Haimen, China) was used to visualize samples. A final staining with hematoxylin was then done. Measurements of the integrated optical density (IOD) of CD31-stained area and blood vessel numbers were done using Image-Pro Plus version 6.0 (Media Cybernetics, Silver Spring, MD) with 3 randomly selected areas used to calculate the IOD/area and the numbers of blood vessel for each sample.

### Flow cytometry analysis of peripheral T cells

Tail veins of the recipient rats were cannulated to extract 50 μL peripheral blood that was then incubated for 15 min in heparinized Eppendorf tubes with red blood cell lysate (Beyotime, Shanghai, China). Then, cells were stained in the dark for 30 min at 4°C with the following antibodies: PerCP-Cy5.5-CD45RA, APC-CD3, PE/Cy7-CD4, PE-CD8a, and V500-CD25 (BD Pharmingen, Franklin Lake, WI, USA). For Treg analysis, Foxp3/Transcription Factor Staining Buffer (eBioscience, San Diego, CA, USA) was first used to fix and permeabilize the cells before they were subjected to incubation with the anti-FoxP3 monoclonal antibody (eBioscience). After being processed, PBS was used to rinse samples twice before being resuspended. Flow cytometry (BD FACSCanto II), BD Diva software, and FlowJo software were used to analyze resultant data.

### Detection of cytokine levels in peripheral blood

Lewis rats recipients were subjected to withdrawal of serum samples which were then maintained at − 80 °C, until further experiments were carried out. These samples were measured for levels of vascular endothelial growth factor (VEGF)-A, tumor necrosis factor (TNF)-α, transforming growth factor-β (TGF-β), and serum interleukin-10 (IL-10) with an enzyme-linked immunosorbent assay kit (R&D Systems, Minneapolis, MN, USA) in compliance to protocols stipulated by the manufacturer.

### Statistical analysis

Data analysis was carried out with the GraphPad Prism 6 software (La Jolla, CA). The log-rank test was used to assess survival rates. Analysis of the single measurable parameters was assessed with the one-way ANOVA test with post hoc analysis conducted using Tukey’s multiple comparison test. For the flow cytometric and anti- and pro-inflammatory cytokines analyses, statistical analysis of all groups was performed using two-way ANOVA with post hoc analysis conducted using Tukey’s multiple comparison test. A *p* value of < 0.05 was considered to reflect statistical significance.

## Result

### Adipose-derived cellular therapies prolong allograft survival

Lewis rat-derived inguinal adipose tissue was used for SVF and ADSC isolation. ADSC were multiplied using protocols that were previously reported [24]. The presence of negatively staining CD45 and positively staining mesenchymal stem cell surface markers CD90 and CD44 confirmed ADSC. ADSC were then stimulated to differentiate into adipogenic, osteogenic, and chondrogenesis lineages (Figure S1).

**Fig. 1:**
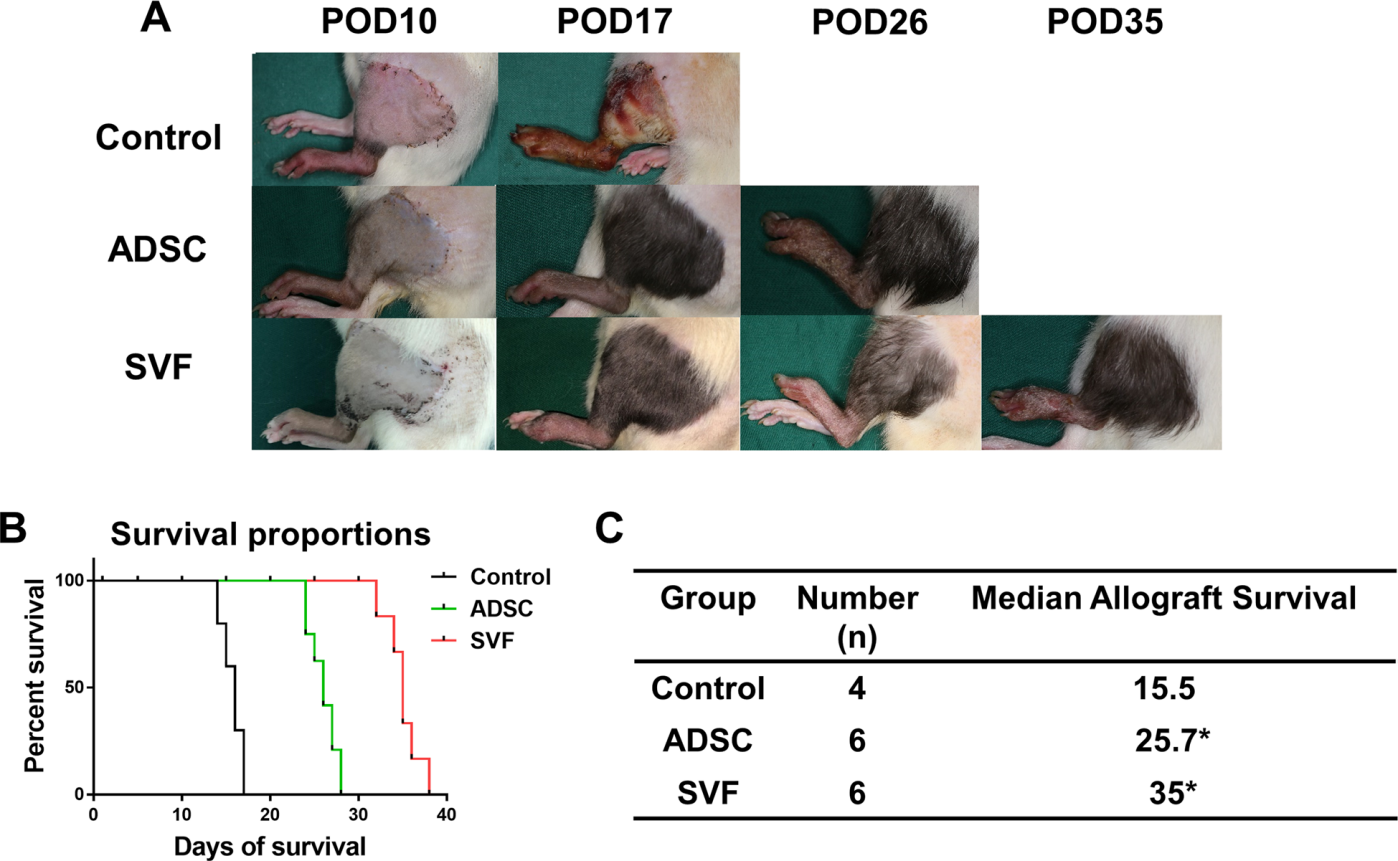
**a** Allograft images in control, ADSC, and SVF groups at post-operative days 10, 17, 26, and 35. **b** Survival rate of control, ADSC, and SVF groups. **c** Survival data summarized in a table. Significant differences are indicated by *P < 0.05

The results showed that hind limb allografts demonstrated obvious symptoms of grade III rejection, including necrosis, exudation, epidermolysis, and histological desquamation at a mean of 15.5 days in the control group. The average survival time of the allografts was 25.7 days in the ADSC treatment group. Allograft recipients treated with SVF ex vivo perfusion survived for a mean of 35 days, indicating a statistically significant increased survival time between the three groups (Fig. 1). Animal models that suffered from surgical failure, such as venous thrombosis, were excluded from statistical analysis.

### Adipose-derived cells alleviate the rejection response of allotransplants

**Fig. 2:**
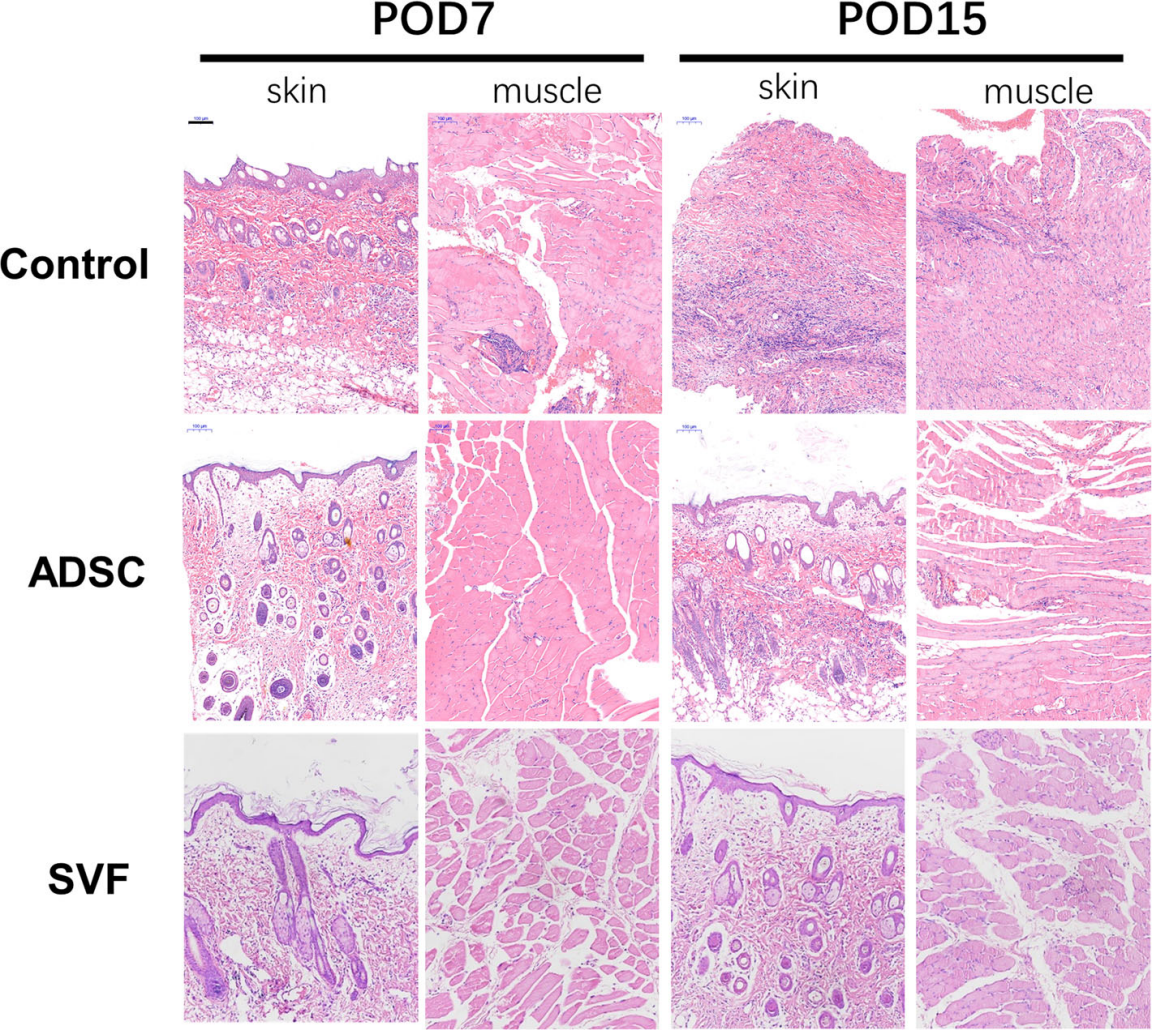
Skin and muscle samples of different groups are stained with hematoxylin and eosin (scale bar = 100 μm)

Daily observations of the transplanted limb were done to watch out for signs of rejection. At 7 and 15 days post-operatively, full-thickness biopsies of donor skin and muscle were obtained. Figure 2 demonstrates that no notably differences between all three groups at 7 days post-operatively. However, those in the control group began to demonstrate deranged muscle and skin tissue architecture and diffuse mononuclear cells infiltration at 15 days post-operatively. In the ADSC group, these changes were present but to a lesser degree. None of these features were noted in the SVF group.

### Robust augmentation of anti-inflammatory cytokine expression modulates immune cell frequencies in blood with adipose-derived cells treatment

**Fig. 3:**
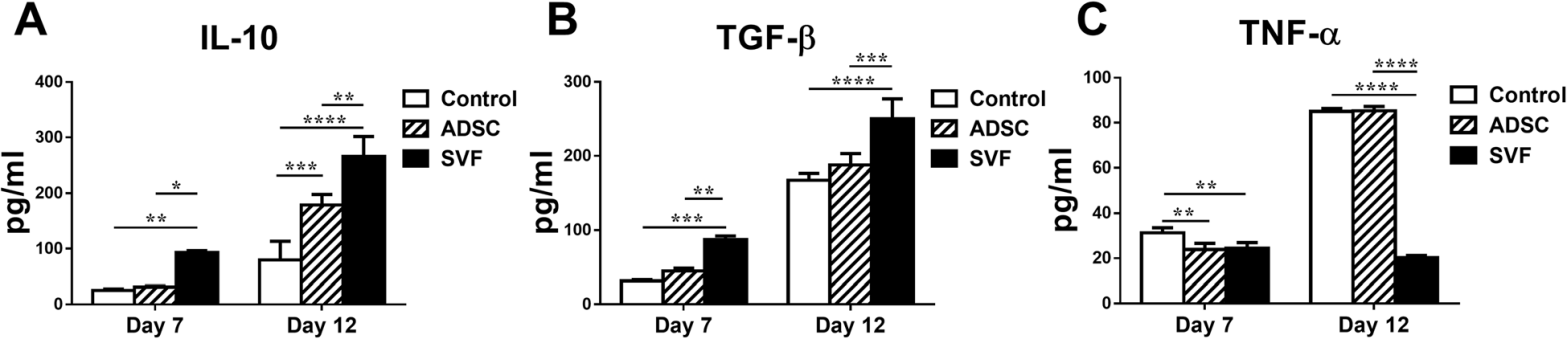
Concentrations of anti-inflammatory and pro-inflammatory cytokines in serum detected by ELISA. **a** IL-10, **b** TGFβ1, and **c** TNF-α (*P < 0.05, **P < 0.01, ***P < 0.001, ****P < 0.0001)

The concentrations of pro-inflammatory cytokine TNF-α and anti-inflammatory cytokines IL-10 and TGF-β in peripheral blood were determined by ELISA on postoperative day 7 and day 12. Those in the ADSC group had raised IL-10 only on day 12 post-procedure while those in the SVF group had raised IL-10 levels noted since as early as day 7 (Fig. 3a). Besides, those in the SVF group also demonstrated raised TGF-β levels on postoperative day 7 and 12 (Fig. 3b). In contrast to the control group, lower TNF-α levels were noted in both the SVF and ADSC-treated groups at 7 days post-operatively while those in the SVF group had persistently low levels of this marker even on day 12 (Fig. 3c).

**Fig. 4:**
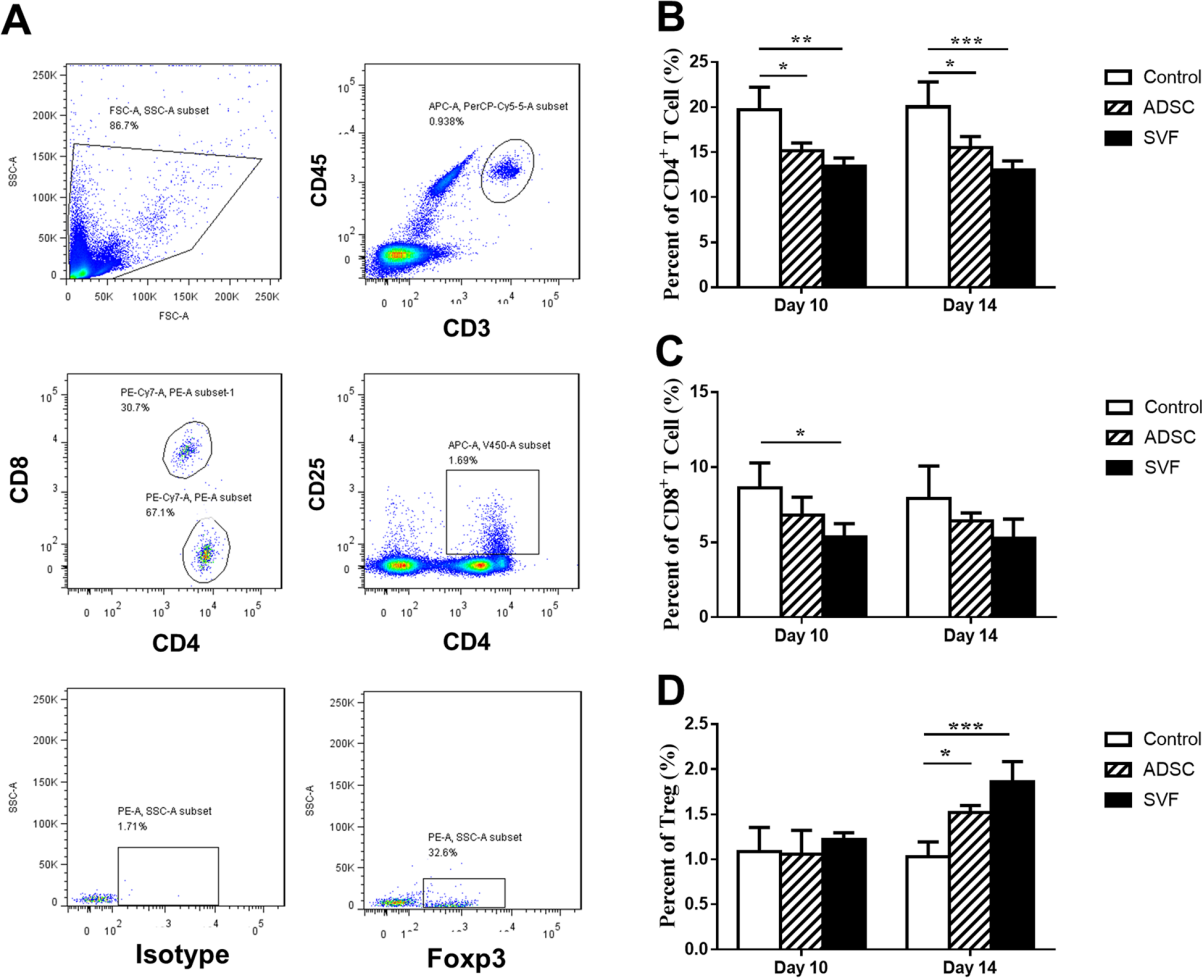
Gating strategy and percentages of T cells. **a** Gating strategy for CD4+, CD8+, and Treg cells. **b** Percentage of CD4+ T cells of three groups at post-operative days 10 and 14. **c** Percentage of CD8+ T cells of three groups at post-operative days 10 and 14. **d** Percentage of Treg cells of three groups at post-operative days 10 and 14 (*P < 0.05, **P < 0.01, ***P < 0.001)

T cell subsets of three groups were measured on day 10 and day 14 post-procedure (Fig. 4). Both SVF and ADSC had lower levels of CD4+ T cells at both the aforementioned time points in contrast to the control group (Fig. 4b). The proportion of CD8+ T cells in SVF treated groups was also noted to be low on postoperative day 10 (Fig. 4c). Both SVF and ADSC groups had higher Treg levels at day 14 post-operatively in contrast to the control group (Fig. 4d).

### SVF treatment promotes vascularization

**Fig. 5:**
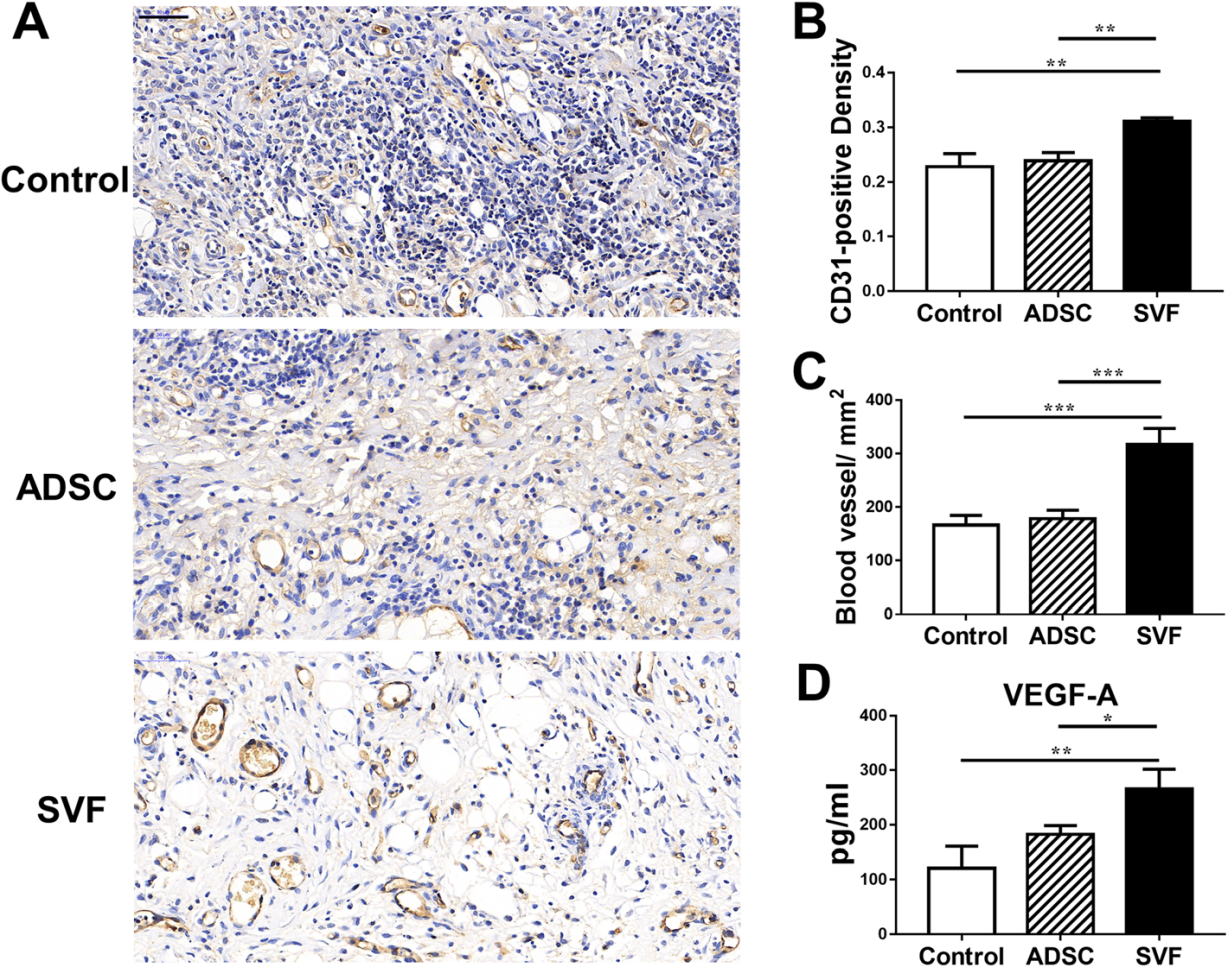
CD31 expression in allografts and VEGF-A expression in serum. **a** Images of allograft skin tissue subjected to immunohistochemical of three groups at post-operative day 7 (scale bar = 50 μm). **b** Quantitative analysis of integrated optical density (IOD) of CD31-stained area. **c** Quantification of blood vessel number in CD31-stained tissue sections. **d** Concentrations of VEGF-A in serum (*P < 0.05, **P < 0.01, ***P < 0.001)

We observed CD31-expressing endothelial cells in immunohistochemical (IHC) stains done on allograft skin tissue at postoperative day 7 (Fig. 5). Stronger CD31 staining was noted in the SVF group in contrast to the control and ADSC group (Fig. 5a). Those in the SVF group also had much larger integrated optical density (IOD) of CD31-stained area and blood vessel numbers compared to the control and ADSC treated groups (Fig. 5b, c). We also tested blood VEGF-A levels at postoperative day 7 and found its expression to be significantly raised in the SVF group compared to the other two groups (Fig. 5d).

## Discussion

Lifelong administration of immunosuppressive drugs after VCA is a major impediment to the benefits of VCA reconstructive surgery. Therefore, the induction and maintenance of donor-specific tolerance towards allografts is an important field of research.

For decades, there have been several studies regarding the use of cellular therapy to promote peripheral tolerance or to modulate local inflammatory immune responses within the graft in transplantation research. Mesenchymal stem cells have been found to possess immunomodulatory effects in VCA. Kuo et al. [25] reported on the benefits of the systemic delivery of ADSC on prolonged survival of swine allografts. Moreover, both Jeong et al. [26] and Plock et al. [27] found out that graft survival time was significantly improved with multiple (as opposed to single dose) post-transplant injections of donor-derived ADSC, an effect that is attributed to increased circulating Treg numbers and reduced allograft infiltration of leukocytes. Our group previously demonstrated the importance of ADSC pre-treatment as an adjunct for improving allograft survival in VCA [18]. This article compares ADSC to SVF pre-treatment in improving allograft survival. Interestingly, we found that the latter was more efficient in enhancing allograft survival. We postulate that this effect is mediated by decreased proliferation and tissue infiltration of lymphocytes as well as modulation of immunomodulatory cytokines IL-10 and TGF-β and increased Treg expression levels. Furthermore, SVF treatment increases the expression of VEGF-A in serum and CD31 in cells of allografts which indicates that SVF could reduce the graft healing time by promoting neovascularization.

A majority of studies focus on immunomodulatory properties regarding ADSC, with a paucity of literature surrounding SVF. ADSC have the advantage of a uniform preparation. However, the ADSC phenotype is easily influenced by their microenvironment [28, 29]. The immunomodulatory properties of ADSC may be lost or seriously diminished during long-term storage [30]. In contrast, freshly isolated SVF do not require in vitro culture and expansion, reducing the risks of contamination and phenotype variation [31]. Moreover, SVF contains a multitude of regulatory immune cells that are involved in the maintenance of immune homeostasis in the adipose tissue microenvironment [32]. These cells improve anti-inflammatory capabilities of other resident cells in adipose tissue, collectively [33]. This impact is likely to continue after isolation, suggesting that it may be essential in maintaining the immunomodulatory functions of the post-transplant environment. Furthermore, SVF treatment could shorten the graft healing time by promoting neovascularization [34, 35]. In sum, we believe that SVF is more appropriate in modulating acute rejection-associated inflammation, thereby inducing peripheral allograft tolerance and vastly improving the overall outcome of VCA recipients.

As a preliminary exploration, this study only contrasts the effectiveness of ADSC and SVF. Future studies regarding the optimal number of injected cells and the time interval of injected cells as well as the relationship between systemic and local treatments are necessary.

## Conclusion

Allograft pre-treatment with SVF enhances allograft survival through suppression of T lymphocyte proliferation and infiltration and augmented immunomodulatory cytokine secretion as well as induction of Treg expression and CD31 and VEGF-A expressions. This treatment modality may be a key adjunct in the introduction and maintenance of immunotolerance in VCA.

## Supplementary Information

**Additional file 1: Figure S1.** Characterization of ADSC. A. Flow cytometric analysis of ADSC, demonstrating proportions of CD90+ (99.5%), CD44+ (99.7%) and CD45+ (1.45%) cells. B. ADSC images as visualized through microscopy (scale bar =100 μm), C. Oil red O-stained adipocytes 3 weeks after induction (scale bar = 40 μm), D. Alizarin red-stained osteocytes 2 weeks after induction (scale bar = 100 μm). E. Alcian blue-stained chondrocytes 4 weeks after induction (scale bar = 50 μm).

## Data Availability

All data generated or analyzed during this study are included in this article.

## Abbreviations

VCA: Vascularized composite allotransplantation
SVF: Stromal vascular fraction
BMSC: Bone marrow-derived stromal cells
ADSC: Adipose-derived stem cells
IHC: Immunohistochemical staining
TNF: Tumor necrosis factor
TGF: Transforming growth factor
IL: Interleukin
VEGF: Vascular endothelial growth factor
IOD: Integrated optical density
