# Supplementary figures and images for: Adipose-derived cellular therapies prolong graft survival in an allogenic hind limb transplantation model
Source: Stem Cell Res Ther. 2021 Jan 29;12:94. doi: 10.1186/s13287-021-02162-7 (PMC7847016; doi:10.1186/s13287-021-02162-7)

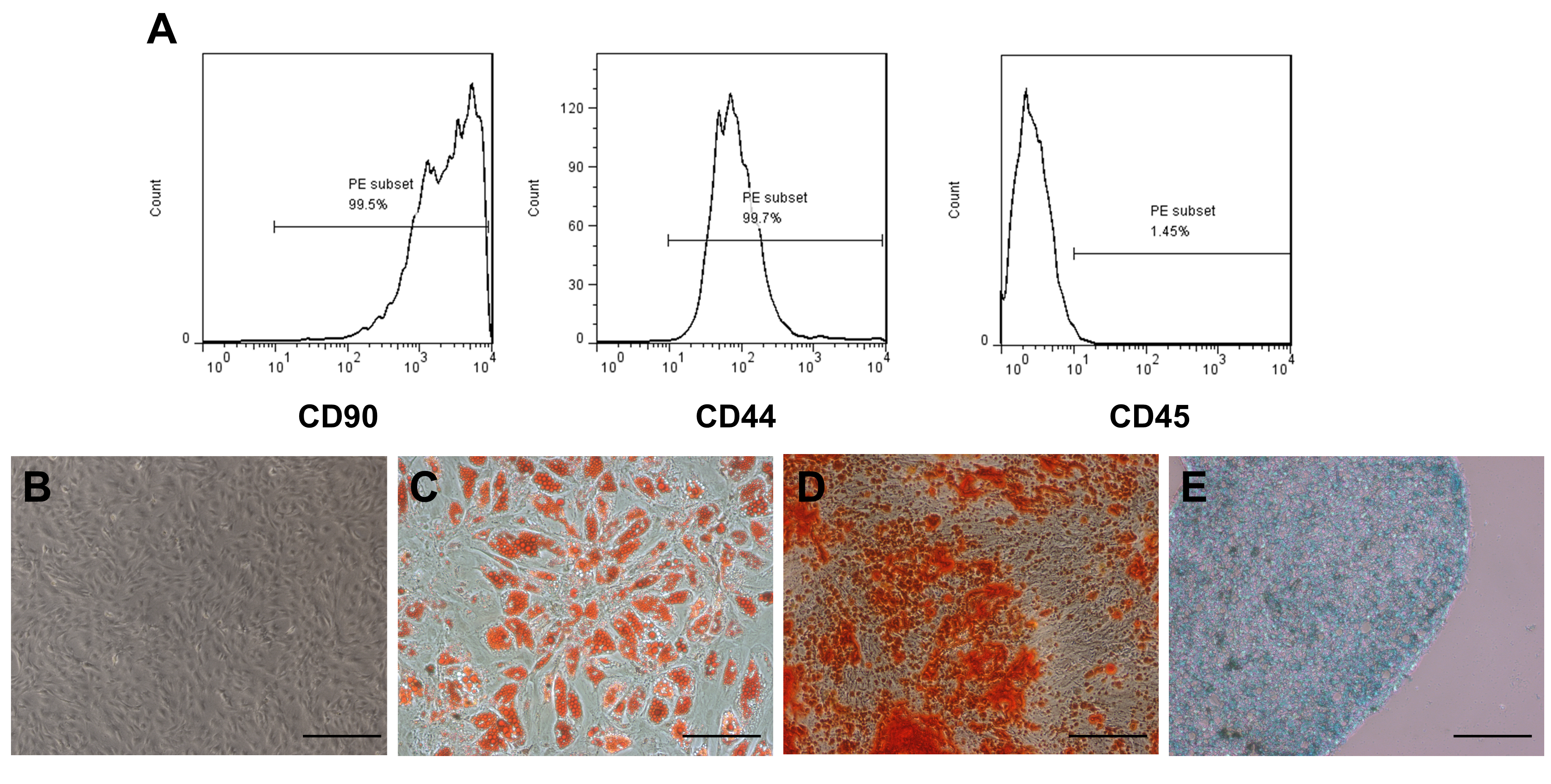

Supplement: Supplementary file 1 — Additional file 1: Figure S1. Characterization of ADSC. A. Flow cytometric analysis of ADSC, demonstrating proportions of CD90+ (99.5%), CD44+ (99.7%) and CD45+ (1.45%) cells. B. ADSC images as visualized through microscopy (scale bar =100 μm), C. Oil red O-stained adipocytes 3 weeks after induction (scale bar = 40 μm), D. Alizarin red-stained osteocytes 2 weeks after induction (scale bar = 100 μm). E. Alcian blue-stained chondrocytes 4 weeks after induction (scale bar = 50 μm). [file 13287_2021_2162_MOESM1_ESM.tif]
